# The Change in Exergaming From Before to During the COVID-19 Pandemic Among Young Adults: Longitudinal Study

**DOI:** 10.2196/41553

**Published:** 2023-05-22

**Authors:** Erin K O'Loughlin, Catherine M Sabiston, Roxy H O'Rourke, Mathieu Bélanger, Marie-Pierre Sylvestre, Jennifer L O'Loughlin

**Affiliations:** 1 Department of Social and Preventive Medicine Centre de recherche du centre hospitalier de l’Université de Montréal Montreal, QC Canada; 2 Faculty of Kinesiology and Physical Education University of Toronto Toronto, ON Canada; 3 Department of Family and Emergency Medicine Université de Sherbrooke Moncton, NB Canada; 4 Department of Family and Emergency Medicine Centre de formation médicale du Nouveau-Brunswick Moncton, NB Canada; 5 School of Public Health Université de Montréal Montreal, QC Canada

**Keywords:** exergaming, active video games, longitudinal study, COVID-19 pandemic, physical activity, serious game, youth, young adult, health promotion, digital health intervention, exergame

## Abstract

**Background:**

Exergaming may be an important option to support an active lifestyle, especially during pandemics.

**Objective:**

Our objectives were (1) to explore whether change in exergaming status (stopped, started or sustained exergaming, or never exergamed) from before to during the COVID-19 pandemic was related to changes in walking, moderate-to-vigorous physical activity (MVPA) or meeting MVPA guidelines and (2) to describe changes among past-year exergamers in minutes per week exergaming from before to during the pandemic.

**Methods:**

A total of 681 participants (mean age 33.6; SD 0.5 years; n=280, 41% male) from the 22-year Nicotine Dependence in Teens (NDIT) study provided data on walking, MVPA, and exergaming before (2017 to 2020) and during (2021) the COVID-19 pandemic. Physical activity (PA) change scores were described by change in exergaming status.

**Results:**

We found that 62.4% (n=425) of the 681 participants never exergamed, 8.2% (n=56) started exergaming during the pandemic, 19.7% (n=134) stopped exergaming, and 9.7% (n=66) sustained exergaming. Declines were observed in all 3 PA indicators in all 4 exergaming groups. The more salient findings were that (1) participants who started exergaming during COVID-19 reported the highest MVPA levels before and during the pandemic and declined the least (mean –35 minutes/week), (2) sustained exergamers reported the lowest MVPA levels during the pandemic (median 66 minutes/week) and declined the most in MVPA (mean change of –92 minutes/week) and in meeting MVPA guidelines (–23.6%). During the pandemic, starting exergamers reported 85 minutes of exergaming per week and sustained exergamers increased exergaming by a median 60 minutes per week.

**Conclusions:**

Although starting and sustaining exergaming did not appear to help exergamers maintain prepandemic PA levels, exergaming can contribute a substantial proportion of total PA in young adults and may still represent a useful option to promote PA during pandemics.

## Introduction

Many studies conducted early on during the COVID-19 pandemic suggest widespread decreases in physical activity (PA) related to public health lockdowns and restrictions [1-5]. Specifically, working from home was associated with declines in active transport, and the closure of gyms and recreational centers limited access to and opportunities for PA [1-5]. Conversely, some pandemic-related changes may have facilitated PA [6]. With less time needed for commuting to and from work, leisure time may have increased for some, thus affording more time to engage in PA [6].

A review on PA during COVID-19 suggested that “positive technology” (defined as a “scientific and applied approach to the use of technology for improving the quality of our personal experience”) [7,8] represented a popular option to support or maintain an active lifestyle during the pandemic [9,10]. Use of technology has been suggested by the World Health Organization (WHO) as one alternative to stay physically active during confinements [11], and many appear to have turned to positive technology during the COVID-19 outbreak to maintain or increase PA [7,9].

Exergaming (or active video gaming), one form of positive technology that engages users in PA, can be played using gaming consoles, mobile devices, virtual reality, personal computers, and specific types of exercise equipment (eg, stationary bikes and treadmills with interactive screens) [7,10,12]. It offers a PA option that is both enjoyable and motivating because of self-monitoring options, gamification, and user interface technology [7,13]. Pokémon Go, for example, is a notably popular exergame that increased PA in both men and women of all ages and weights, even reaching low-activity populations [12]. Exergaming offers personalized, convenient activity options, as well as the possibility of playing with other users online, which, in addition to decreasing sedentary time, may decrease feelings of social isolation during pandemic-related restrictions [13-15]. Exergaming can help people cope with mental health issues such as anxiety [9,16,17], and it can support those with chronic disease, such as Parkinson diseases [17,18], poststroke sequelae [19], arthritis [20], and brain injuries [21], in maintaining PA levels in home-based rehabilitation situations during pandemics.

Before the COVID-19 pandemic, the prevalence of weekly exergaming among adolescents and young adults ranged from 18% to 43% in Canada [22-25]. However, it is not clear whether the prevalence changed during the COVID-19 pandemic. Further, no longitudinal studies have assessed whether exergaming was associated with changes in PA levels during the pandemic. Because future pandemics are possible, understanding exergaming behavior during the COVID-19 pandemic could shed light on potential benefits and challenges of using technology to encourage PA during lockdowns. Further, stressful times can encourage positive behavior change (eg, taking up exergaming) that may endure postpandemic [13,17,26]. Therefore, building the evidence base in this realm could inform public health decisions related to promoting PA during a pandemic.

The objectives of this study were to (1) explore whether a change in exergaming status (stopped, started or sustained exergaming, or never exergamed) from before to during the COVID-19 pandemic related to changes in walking, minutes of moderate-to-vigorous physical activity (MVPA), and meeting MVPA guidelines. We were particularly interested in ascertaining whether starting to exergame or sustaining exergaming were associated with maintaining pre–COVID-19 PA levels of walking, MVPA, or meeting MVPA guidelines. We also aimed to (2) describe changes in minutes of exergaming per week among past-year exergamers from before to during the COVID-19 pandemic.

## Methods

### Study Sample

Data were drawn from the Nicotine Dependence in Teens (NDIT) study [27], an ongoing 23-year longitudinal study that aims to describe the natural course of nicotine dependence in youth, but also collects data on a wide range of sociodemographic, substance use, psychosocial, lifestyle, and health-related variables. A detailed description of the NDIT study methods has been published previously [27]. Relevant to this study, data for 799 participants were collected in cycle 23 between January 2017 to March 2020 (n=551, 69% of participants completed the questionnaire in 2017; n=152, 19% in 2018; n=88, 11% in 2019; and n=8, 0.8% in 2020). Data were available for 722 participants in cycle 24, which was conducted online during the COVID-19 pandemic, from December 2020 to February 2021. Participants could use their mobile phone, tablet, or personal computer to complete the questionnaire. A total of 681 participants in cycle 23 (85%) also provided data in cycle 24 and were retained for this analysis. Participants received a CAD $50 (US $36.97) gift card or e-transfer in each cycle to cover any costs associated with their participation.

### Ethical Approval

The study procedures were approved by the Montreal Department of Public Health Ethics Review Committee (2007–2384), the McGill University Faculty of Medicine Institutional Review Board (2017–6895), and the Ethics Research Committee of the Centre de Recherche du Centre Hospitalier de l’Université de Montréal (2021-9385, 20.278-YP). Parental consent was obtained at NDIT inception. Participants could legally provide consent in the post–high school data collections because they had all attained the age of 18 years.

### Study Variables

#### PA Variables

Data on number of minutes per week walking, moderate physical activity, and vigorous physical activity were collected using the short form of the International Physical Activity Questionnaire (IPAQ-SF) [28,29]. The IPAQ-SF is used in cross-national monitoring of PA and demonstrates low to moderate reliability and validity against device-based PA measures [30]. Participants reported the number of days on which they had engaged in each type of PA over the past week and the average number of minutes per bout. The recommended truncation protocol [29] was used to exclude unrealistic values and reduce improbable PA scores. Number of minutes walking per week was calculated as the number of days walking per week multiplied by the number of minutes per bout. Number of moderate PA minutes per week was computed as the number of days of moderate PA per week multiplied by the number of minutes per bout, and the number of vigorous PA minutes per week was computed as the number of days of vigorous PA per week multiplied by the number of minutes per bout.

#### Calculation of MVPA

Number of MVPA minutes per week was calculated by adding the number of moderate PA minutes per week and number of vigorous PA minutes per week.

#### Meeting MVPA Guidelines

Participants were categorized as meeting MVPA guidelines (yes or no) if they had engaged in MVPA for ≥150 minutes per week [31].

#### Exergaming Status

Using data from cycles 23 and 24, participants were categorized into 4 groups based on their exergaming behavior over time using the following item: “In the past 12 months, how often did you exergame using consoles, or using your cellphone and/or a mobile app?” Response choices included never, less than once a month, 1 to 3 times a month, 1 to 6 times per week, and every day. In both cycles 23 and 24, participants were categorized as exergamers if they chose any response option other than “never.” The four groups included (1) never-exergamers (ie, participants who did not report exergaming in cycles 23 or 24); (2) stopping exergamers (ie, participants who reported exergaming in cycle 23 but not in cycle 24); (3) starting exergamers (ie, participants who reported exergaming in cycle 24 but not in cycle 23); and (4) sustaining exergamers (ie, participants who exergamed in both cycles 23 and 24).

#### Minutes Exergaming per Week

Items measuring the number of minutes exergaming per week were modeled on the IPAQ-SF [22,23,25]. Participants who reported past-year exergaming were asked: “Did you exergame using consoles such as Nintendo Wii, XBOX ONE Kinect, Sony Play Station Move, Sony Eye Toy: Kinetic, or using your cellphone and/or a mobile app in the past 30 days? (ex. ZOMBIES, RUN!, Nike+ Running App, Fit Freeway, Pokémon Go)?” Response options were yes or no. Participants who responded no were assigned a score of 0 for minutes exergaming per week. Those who responded yes were asked how many days per week they exergamed (the response option was from 1 to 7 days) and how many minutes they played on average during each bout in the past month (responses were open-ended). The total number of minutes exergaming per week was calculated as the number of days exergaming per week multiplied by the average number of minutes per bout.

#### Exergaming Intensity

Usual intensity of exergaming was measured by the following question: “What was your usual physical effort during play?” Response choices included light, moderate, and vigorous.

#### Sociodemographic Characteristics

Sociodemographic characteristics included age, sex, whether the participant was university educated (yes or no), whether their mother was university educated (yes or no), and whether they were French speaking (yes or no), were born in Canada (yes or no), had an annual household income <CAN $50,000 (US $36,966; yes or no), lived alone (yes or no), lived with children (yes or no), and were employed (yes or no).

### Data Analyses

Descriptive statistics were used to compare sociodemographic and PA-related characteristics (ie, walking, MVPA, and exergaming) of participants retained in the analytic sample (ie, those who provided data in cycles 23 and 24) versus not retained (ie, those who provided data in cycle 23 but not cycle 24). Based on skewness and kurtosis, walking, MVPA, and exergaming minutes per week were not normally distributed. Therefore, the median (IQR) is reported for these variables. The proportion of participants that met MVPA guidelines is reported as a percentage.

Change scores were computed by subtracting number of minutes per week for each PA indicator (including exergaming) in cycle 23 from minutes per week in cycle 24. Because the distributions for the change scores were relatively normal in each of the 4 groups defined by exergaming status as well as in the sample overall, we report mean (SD) change scores. We report the median (IQR) for the change in minutes exergaming per week because the data were not normally distributed. Data were analyzed using SPSS (version 20.0; IBM Corp).

## Results

### Participants Retained Versus Not Retained

Table 1 compares the sociodemographic and PA-related characteristics of participants retained and not retained (ie, as measured in cycle 23). Lower proportions of those retained were male and French speaking. In addition, they reported fewer minutes per week walking and engaging in MVPA than participants who were not retained.

The mean age of the 681 participants retained for analysis was 33.6 (SD 0.5) years in cycle 24; 41% (n=280) were male, 95% (n=647) were born in Canada, 33% (n=225) were French speaking, 21% (n=143) had an annual household income of <CAN $50,000 (US $36,966), 15% (n=102) lived alone, 51% (n=347) lived with children, 83% (n=565) reported being employed, and 36% (n=245) met MVPA guidelines. The median (IQR) minutes per week during COVID-19 was 120 (45-240) for walking and 80 (0-240) for MVPA. Participants who exergamed during COVID-19 (n=122) reported a median (IQR) of 80 (9-240) minutes of exergaming per week.

Of 681 participants retained for analysis, 425 (62.4%) were never-exergamers, 56 (8.2%) started exergaming in cycle 24 (during the pandemic), 134 (19.7%) stopped exergaming in cycle 24, and 66 (9.7%) sustained exergaming. Among the 200 exergamers in cycle 23, 66 (33%) sustained exergaming in cycle 24, and 134 (67%) stopped exergaming.

Table 2 reports the median number of minutes walking per week, median number of minutes engaged in MVPA per week, and the percentage of participants who met MVPA guidelines before and during the COVID-19 pandemic. In addition to reporting values for the sample overall, data are provided for the 4 groups defined by exergaming behavior over time.

The data suggest declines over time in all 3 PA indicators in the sample overall (Table 2). Median minutes walking per week declined from 175 before COVID-19 to 120 during COVID-19, with a mean decline of 58 minutes per week. MVPA declined from a median 130 to 80 minutes per week among the participants overall, with a mean decline of 49 minutes per week. The proportion of participants that met MVPA guidelines declined from 48.3% (n=326) before the pandemic to 36% (n=245) during the pandemic, an absolute decline of 12.3%.

**Table 1:** Characteristics of participants retained and not retained for analysis from the Nicotine Dependence in Teens study (Montreal, Quebec, 2017-2021). Data were drawn from cycle 23^a^.

Characteristics^b^	Total (n=799)	Retained in cycle 24 (n=681)	Not retained in cycle 24 (n=118)
Age (years), mean (SD)	30.6 (1.0)	30.5 (1.0)	31.0 (1.1)
Male, n (%)	351 (43.9)	280 (41.1)	71 (60.2)
Born in Canada, n (%)	799 (93.7)	642 (94.3)	107 (90.7)
Mother is university educated, n (%)	332 (46.2)	283 (45.6)	49 (50)
French speaking, n (%)	245 (30.7)	199 (29.2)	46 (39)
Walking (minutes/week), median (IQR)	175(60-350)	175 (60-350)	200 (70-420)
MVPA^c^ (minutes/week), median (IQR)	135 (0-330)	135 (0-303)	180 (20-450)
Met MVPA guidelines, n (%)	387 (49.5)	326 (48.9)	61 (53)
Exergaming (minutes/week), median (IQR)^c^	0 (0-0)	0 (0-0)	0 (0-83)

^a^Totals may differ due to missing data.

^b^Includes only past-year exergamers.

^c^MVPA: moderate-to-vigorous physical activity.

**Table 2:** Number of minutes walking per week, number of minutes engaged in moderate-to-vigorous physical activity per week, and percentage of participants who met moderate-to-vigorous physical activity guidelines from before to during the COVID-19 pandemic in 4 groups defined by consistency in exergaming behavior over time (Nicotine Dependence in Teens study, Montreal, Canada, 2017-2021)^a^.

			Before COVID-19 (cycle 23^b^)		During COVID-19 (cycle 24^c^)		Change between cycles 23 and 24
**Walking (minutes/week), median (IQR)**							
	Overall (n=681)	175 (60-350)		120 (45-240)		–58 (310)^d^	
	Never exergamed (n=424)	175 (60-315)		120 (45-240)		–48 (298)^d^	
	Started exergaming (n=56)	163 (90-420)		195 (40-420)		–38 (372)^d^	
	Stopped exergaming (n=134)	178 (88-420)		120 (60-210)		–97 (279)^d^	
	Sustained exergaming (n=66)	140 (30-258)		90 (40-188)		–56 (380)^d^	
**MVPA^e^ (minutes/week), median (IQR)**							
	Overall (n=681)	130 (0-310)		80 (0-240)		–49 (342)^d^	
	Never exergamed (n=424)	120 (0-300)		80 (0-240)		–41 (336)^d^	
	Started exergaming (n=56)	210 (40-420)		90 (4-394)		–35 (395)^d^	
	Stopped exergaming (n=134)	180 (41-360)		80 (0-275)		–58 (306)^d^	
	Sustained exergaming (n=66)	180 (0-360)		66 (0-221)		–92 (405)^d^	
**Met MVPA guidelines, n (%)**							
	Overall (n=681)	326 (48.3)		245 (36)		–12.3^f^	
	Never exergamed (n=424)	186 (44.8)		155 (36.6)		–8.2^f^	
	Started exergaming (n=56)	31 (57.1)		21 (37.5)		–19.6^f^	
	Stopped exergaming (n=134)	74 (55.6)		48 (35.8)		–19.8^f^	
	Sustained exergaming (n=66)	35 (55.4)		21 (31.8)		–23.6^f^	

^a^Totals may differ due to missing data.

^b^Data were collected from January 2017 to March 2020 (mean age 30.6 years).

^c^Data were collected from December 2020 to June 2021 (mean age 33.6 years).

^d^These values are the mean (SD), since the change scores were normally distributed.

^e^MVPA: moderate-to-vigorous physical activity.

^f^These values represent the absolute decline in percentage.

### Change in Walking Across Exergaming Groups

Among the 4 groups defined by exergaming status, patterns of interest in walking from before to during COVID-19 (Table 2) show that those who started exergaming reported the highest level of walking during the pandemic (a median 195 minutes per week), and in addition, they declined the least (they had a mean decline of 38 minutes per week, compared to 58 minutes among all participants). Those who stopped exergaming had the highest level of walking before COVID-19 (median 178 minutes per week) and declined the most (their mean minutes walking per week declined by 97 minutes). Finally, sustained exergamers reported the lowest median levels of walking both before and during the pandemic; the mean decline in minutes walking per week in this group (–56 minutes per week) was similar to the mean decline among participants overall (–58 minutes per week).

### Change in MVPA Across Exergaming Groups

Patterns of interest in MVPA in the 4 exergaming status groups (Table 2) show that never-exergamers reported the lowest MVPA levels before the COVID-19 pandemic (a median 120 minutes per week). Participants who started exergaming during the pandemic reported the highest MVPA levels both before and during the COVID-19 pandemic, and they declined the least (Table 2). Their mean MVPA change score was –35 minutes MVPA per week compared to –49 minutes among participants overall. Finally, sustained exergamers reported the lowest MVPA levels during the pandemic (a median 66 minutes per week) and they declined the most. Their mean MVPA change score was –92 minutes per week.

### Change in Meeting MVPA Guidelines Across Exergaming Groups

Compared to the other 3 groups, a higher proportion of participants who started to exergame during the COVID-19 pandemic met MVPA guidelines both before and during the COVID-19 pandemic. Although the proportion of sustained exergamers that met MVPA guidelines was similar to the other 2 exergaming groups before COVID-19, this group reported the lowest proportion during COVID-19 and the largest decline over time (–23.6%). The proportion of participants that met MVPA guidelines was lowest among never-exergamers before COVID-19 (186/424, 44.8%), and this group had the lowest decline over time (–8.2%).

### Change in Minutes Exergaming Among Exergamers

Table 3 reports change in exergaming minutes per week among sustained exergamers, participants who started exergaming, and participants who stopped exergaming. Participants who started exergaming reported a median of 85 minutes of exergaming per week during the COVID-19 pandemic. Sustained exergamers also increased exergaming by a mean 90.5 minutes per week (Table 3). Light intensity exergaming was reported by 65% (47/72) of exergamers in cycle 23 and 59% (55/94) of exergamers in cycle 24. Moderate or vigorous exergaming was reported by 35% (25/72) of exergamers in cycle 23 and 42% (39/94) in cycle 24.

**Table 3:** Number of minutes per week engaged in exergaming before and during the COVID-19 pandemic in groups defined by consistency in exergaming behavior over time (Nicotine Dependence in Teens study, Montreal, Quebec, 2017-2021).

	Exergaming (minutes/week), median (IQR)		
	Before COVID-19 (cycle 23^a^)	During COVID-19 (cycle 24^b^)	Change between cycles 23 and 24
Started exergaming (n=56)	0 (0 to 0)	85 (0 to 240)	85 (0 to 240)
Stopped exergaming (n=134)	0 (0 to 0)^c^	0 (0 to 0)	0 (0 to 0)
Sustained exergaming (n=66)	0 (0 to 63)^c^	68 (14 to 229)	60 (–4 to 188)

^a^Data were collected from January 2017 to March 2020 (mean age 30.6 years).

^b^Data were collected from December 2020 to June 2021 (mean age 33.6 years).

^c^Participants who reported past-year exergaming but 0 minutes exergaming in the past 30 days were assigned a score of 0 minutes exergaming per week.

## Discussion

### Overall Findings

Overall, the percentage of participants meeting MVPA guidelines, the number of minutes walked, and the number of minutes engaged in MVPA per week declined from before to during the pandemic regardless of exergaming status [6]. Our data suggest that sustained exergamers were not more active than never-exergamers during the COVID-19 pandemic and, in fact, appeared to be less active. In contrast, those who started exergaming reported the highest levels of walking and MVPA during COVID-19, although, as in all groups, the change scores suggest declines on average in both activities. Thus, it appears that even if exergaming encourages PA during periods of confinement, in and of itself exergaming may not be sufficient to help exergamers maintain prepandemic PA levels. It is possible, however, that without exergaming the declines observed among sustained exergamers could have been steeper.

### Never-Exergamers

Never-exergamers reported declines of 48 minutes per week in walking and 41 minutes per week in MVPA from before to during the COVID-19 pandemic. Of the 4 exergaming groups studied, never-exergamers reported the lowest levels of MVPA and were least likely to meet MVPA guidelines before the pandemic. The relatively minor changes in PA in this group during COVID-19 may reflect a general disinterest in PA (so that closure of gyms and recreational centers during the pandemic made little difference to their PA levels). It is possible that level of motivation for PA remained low among these individuals during the pandemic, such that they did not benefit from any increases in time available for PA due to containment measures. It is also possible that this group had established (lower) PA levels and patterns that they were content with and that they felt little need to explore different ways of building PA into their routines, regardless of the pandemic context.

### Stopped Exergaming

Among the 4 groups defined by exergaming status, those who stopped exergaming declined the most in walking (–97 minutes/week) from before to during the COVID-19 pandemic. Although stopping exergaming in this group was possibly related to COVID-19, the middle to early 30s is often a turbulent time during the life course marked by numerous important transitions as people complete their education, enter the workforce, and begin their own families [32]. Rather than being a consequence of the COVID-19 pandemic, exergaming may be a form of PA that is dropped during these life transitions to make way for engaging in new roles and activities. Alternatively, exergaming could be an intermittent activity [32] linked to the release of new games or consoles, and the lack of new releases during the pandemic could be among the reasons that participants stopped exergaming. Exergaming may also be a “transferable” PA, such that it is used to experiment with a new PA (eg, to begin jogging), which is then continued independently of the exergaming component. Finally, frustration with technical glitches, concerns with online privacy [33], reduction in enjoyment and game immersion (ie, how a videogame draws a player into the game) [33,34], and price have also been cited as reasons for stopping exergaming [33,34]. Qualitative studies may be helpful in identifying the reasons why exergamers in their 30s choose to stop exergaming during pandemics.

### Sustained Exergaming

Of the 4 exergaming groups, sustained exergamers reported the lowest levels of walking both before and during the pandemic and the lowest levels of MVPA during the pandemic. Previous research suggests that although exergamers do not necessarily have higher PA levels than never-exergamers and may not be interested in traditional PA, they do understand the importance of movement and turn to exergaming for their PA [22,25,35]. Sustained exergamers may have continued to play games such as Pokémon Go during the COVID-19 pandemic. There is evidence that 2020 was the most profitable year ever for the Pokémon Go enterprise [36], suggesting that many exergamers continued to play this outdoor game during COVID-19. Zombies, Run! also had an increase of 2 million users during COVID-19 [37]. Further, Ellis et al [38] reported that among 2004 young adult gamers (aged 30.5 years on average), Pokémon Go and Harry Potter: Wizards Unite were played frequently during the pandemic to maintain exercise levels and for social connection. Many participants reported that Pokémon Go was “the one thing that keeps me going outdoors and moving each day” [34]. Ellis et al [38] also reported that those who exergamed during the COVID-19 pandemic did so to distract themselves, escape from reality for a short period of time, occupy themselves, and manage their mental health.

Sustained exergamers reported a median of 68 minutes per week of exergaming, an important contribution to overall PA. Exergaming is not usually dependent on fitness or recreational facilities outside the home; therefore, this group may not have been overly affected by pandemic-related lockdowns. While the change in walking levels among sustained exergamers was not as marked as among those who stopped exergaming, their decline in MVPA was substantial (–92 minutes per week). It is possible that sustained exergamers participated in other modes of PA before the pandemic that were not replaced during the pandemic. Minutes exergaming per week among sustained exergamers did increase (as also reported in the Ellis et al study), but this was not enough to replace MVPA lost in other PA modes.

### Started Exergaming

Of the 4 exergaming groups, those who started exergaming during the pandemic reported the highest MVPA levels before the pandemic (210 minutes/week) and the smallest decrease in MVPA (–35 minutes/week) during the pandemic. This group may have started using exergaming to compensate for activities they could no longer participate in during COVID-19. In addition to compensating for the loss of PA during the pandemic, video games may have helped with the loss of social interactions, which might have been a particularly salient loss in this group [7,14,38,39]. Whatever the underpinning, starting exergaming during the pandemic likely reflects relatively high levels of “physical literacy,” which represents the motivation and financial resources to search out new forms of accessible PA [40]. Alternatively, or in addition, this group may also have been particularly sensitive to public health messaging that recommended maintaining PA levels during the pandemic.

### Future Research

More research is needed to examine fluctuations in exergaming behaviors over time, especially during such challenging times as COVID-19. Qualitative research may help identify reasons for stopping, starting, or continuing to exergame during pandemics. This study did not identify types of exergaming engaged in, who participants exergamed with, or the context (ie, indoors or outdoors) in which they exergamed. These areas could be important avenues to explore to better inform recommendations for starting exergaming or for maintaining previous levels of exergaming during pandemics, as well as in general.

### Limitations

Limitations of this study include that self-report IPAQ-SF data are subject to overreporting, although Lee et al [28] suggest that because of its established reliability, the IPAQ-SF can be used with care in repeated-measures studies. There was loss to follow-up between cycle 23 and cycle 24, which may relate to competing interests in the busy lives of young people in emerging adulthood. Selection bias related to this loss to follow-up could have attenuated our estimates, although the small number of sociodemographic differences between those retained and not retained for analysis mitigates this concern somewhat. Use of a purposive sample of schools in the NDIT study may limit the generalizability of the findings. Finally, we were unable to categorize exergamers according to more refined categories of exergaming intensity and frequency, and this may have led to misclassification of some exergamers. Future studies should use more refined measures of exergaming to enable more accurate categorization of exergamers.

### Conclusion

Although it may support PA at home during periods of confinement, starting or sustaining exergaming did not appear to be enough to maintain prepandemic PA levels in this population-based sample of young adults. However, the data suggest that exergaming can contribute a substantial proportion of total PA in young adults and may still represent a useful option to promote PA during pandemics.

## Abbreviations

IPAQ-SF: International Physical Activity Questionnaire–Short Form
MVPA: moderate-to-vigorous physical activity
NDIT: Nicotine Dependence in Teens
PA: physical activity
WHO: World Health Organization

## References

[1] Stockwell S, Trott M, Tully M, Shin J, Barnett Y, Butler L, McDermott D, Schuch F, Smith L (2021). Changes in physical activity and sedentary behaviours from before to during the COVID-19 pandemic lockdown: a systematic review. BMJ Open Sport Exerc Med.

[2] Polero P, Rebollo-Seco C, Adsuar JC, Pérez-Gómez Jorge, Rojo-Ramos J, Manzano-Redondo F, Carlos-Vivas J, Garcia-Gordillo (2020). Physical activity recommendations during COVID-19: narrative review. Int J Environ Res Public Health.

[3] Caputo EL, Reichert FF (2020). Studies of physical activity and COVID-19 during the pandemic: A scoping review. J Phys Act Health.

[4] Violant-Holz V, Gallego-Jiménez M Gloria, González-González Carina S, Muñoz-Violant Sarah, Rodríguez Manuel José, Sansano-Nadal O, Guerra-Balic M (2020). Psychological health and physical activity levels during the COVID-19 pandemic: A systematic review. Int J Environ Res Public Health.

[5] Ammar A, Brach M, Trabelsi K, Chtourou H, Boukhris O, Masmoudi L, Bouaziz B, Bentlage E, How D, Ahmed M, Müller Patrick, Müller Notger, Aloui A, Hammouda O, Paineiras-Domingos LL, Braakman-Jansen A, Wrede C, Bastoni S, Pernambuco CS, Mataruna L, Taheri M, Irandoust K, Khacharem A, Bragazzi NL, Chamari K, Glenn JM, Bott NT, Gargouri F, Chaari L, Batatia H, Ali GM, Abdelkarim O, Jarraya M, Abed KE, Souissi N, Van Gemert-Pijnen L, Riemann BL, Riemann L, Moalla W, Gómez-Raja Jonathan, Epstein M, Sanderman R, Schulz SV, Jerg A, Al-Horani R, Mansi T, Jmail M, Barbosa F, Ferreira-Santos F, Šimunič Boštjan, Pišot Rado, Gaggioli A, Bailey SJ, Steinacker JM, Driss T, Hoekelmann A (2020). Effects of COVID-19 home confinement on eating behaviour and physical activity: results of the ECLB-COVID19 international online survey. Nutrients.

[6] O'Loughlin EK, Riglea T, Sylvestre M, Pelekanakis A, Sabiston CM, Bélanger Mathieu, O'Loughlin JL (2022). Stable physical activity patterns predominate in a longitudinal study of physical activity among young adults in Canada from before to during the COVID-19 pandemic. Prev Med Rep.

[7] Riva G, Mantovani F, Wiederhold BK (2020). Positive technology and COVID-19. Cyberpsychol Behav Soc Netw.

[8] Bentlage E, Ammar A, How D, Ahmed M, Trabelsi K, Chtourou H, Brach M (2020). Practical recommendations for maintaining active lifestyle during the COVID-19 pandemic: A systematic literature review. Int J Environ Res Public Health.

[9] Dwyer MJ, Pasini M, De Dominicis S, Righi E (2020). Physical activity: Benefits and challenges during the COVID-19 pandemic. Scand J Med Sci Sports.

[10] Hammami A, Harrabi B, Mohr M, Krustrup P (2020). Physical activity and coronavirus disease 2019 (COVID-19): specific recommendations for home-based physical training. Manag Sport Leis.

[11] Be active at home during the #COVID19 outbreak. World Health Organization.

[12] Althoff T, White RW, Horvitz E (2016). Influence of Pokémon Go on physical activity: Study and implications. J Med Internet Res.

[13] Chtourou H, Trabelsi K, H'mida C, Boukhris O, Glenn JM, Brach M, Bentlage E, Bott N, Shephard RJ, Ammar A, Bragazzi NL (2020). Staying physically active during the quarantine and self-isolation period for controlling and mitigating the COVID-19 pandemic: A systematic overview of the literature. Front Psychol.

[14] Dobbins S, Hubbard E, Flentje A, Dawson-Rose C, Leutwyler H (2020). Play provides social connection for older adults with serious mental illness: A grounded theory analysis of a 10-week exergame intervention. Aging Ment Health.

[15] Brady S, D'Ambrosio LA, Felts A, Rula EY, Kell KP, Coughlin JF (2020). Reducing isolation and loneliness through membership in a fitness program for older adults: Implications for health. J Appl Gerontol.

[16] Viana RB, Dankel SJ, Loenneke JP, Gentil P, Vieira CA, Andrade MDS, Vancini RL, de Lira CAB (2020). The effects of exergames on anxiety levels: A systematic review and meta-analysis. Scand J Med Sci Sports.

[17] Viana RB, de Lira CAB (2020). Exergames as coping strategies for anxiety disorders during the COVID-19 quarantine period. Games Health J.

[18] Langer A, Gassner L, Flotz A, Hasenauer S, Gruber J, Wizany L, Pokan R, Maetzler W, Zach H (2021). How COVID-19 will boost remote exercise-based treatment in Parkinson's disease: a narrative review. NPJ Parkinsons Dis.

[19] Hoaas H, Andreassen HK, Lien LA, Hjalmarsen A, Zanaboni P (2016). Adherence and factors affecting satisfaction in long-term telerehabilitation for patients with chronic obstructive pulmonary disease: a mixed methods study. BMC Med Inform Decis Mak.

[20] Ambrosino P, Iannuzzi GL, Formisano R, Spedicato GA, D'Abrosca V, Di Gioia L, Di Minno MND, Pappone N (2020). Exergaming as an additional tool in rehabilitation of young patients with rheumatoid arthritis: A pilot randomized controlled trial. Games Health J.

[21] Ambrosino P, Fuschillo S, Papa A, Di Minno MND, Maniscalco M (2020). Exergaming as a supportive tool for home-based rehabilitation in the COVID-19 pandemic era. Games Health J.

[22] O'Loughlin EK, Dugas EN, Sabiston CM, O'Loughlin JL (2012). Prevalence and correlates of exergaming in youth. Pediatrics.

[23] O'Loughlin E, Sabiston CM, Kakinami L, McGrath JJ, Consalvo M, O'Loughlin JL, Barnett TA (2020). Development and validation of the Reasons to Exergame (RTEX) scale in young adults: exploratory factors analysis. JMIR Serious Games.

[24] O’Loughlin E (2019). Contribution of exergaming behaviour to physical activity: Toward better understanding the role of motivation.. Concordia University.

[25] Kakinami L, O'Loughlin EK, Dugas EN, Sabiston CM, Paradis G, O'Loughlin J (2015). The association between exergaming and physical activity in young adults. J Phys Act Health.

[26] Stewart SF, Ogden J (2021). Motivating or stigmatising? The public health and media messaging surrounding COVID-19 and obesity: a qualitative think aloud study. Health Educ.

[27] O'Loughlin J, Dugas EN, Brunet J, DiFranza J, Engert JC, Gervais A, Gray-Donald K, Karp I, Low NC, Sabiston C, Sylvestre M, Tyndale RF, Auger N, Auger N, Mathieu B, Tracie B, Chaiton M, Chenoweth MJ, Constantin E, Contreras G, Kakinami L, Labbe A, Maximova K, McMillan E, O'Loughlin EK, Pabayo R, Roy-Gagnon M, Tremblay M, Wellman RJ, Hulst A, Paradis G (2015). Cohort Profile: The Nicotine Dependence in Teens (NDIT) Study. Int J Epidemiol.

[28] Lee PH, Macfarlane DJ, Lam TH, Stewart SM (2011). Validity of the International Physical Activity Questionnaire Short Form (IPAQ-SF): a systematic review. Int J Behav Nutr Phys Act.

[29] Forde C (2018). Scoring the International Physical Activity Questionnaire (IPAQ). Trinity College Dublin.

[30] Dyrstad SM, Hansen BH, Holme IM, Anderssen SA (2014). Comparison of self-reported versus accelerometer-measured physical activity. Med Sci Sports Exerc.

[31] Piercy KL, Troiano RP, Ballard RM, Carlson SA, Fulton JE, Galuska DA, George SM, Olson RD (2018). The Physical Activity Guidelines for Americans. JAMA.

[32] Sylvestre M, Dinkou GDT, Naja M, Riglea T, Pelekanakis A, Bélanger Mathieu, Maximova K, Mowat D, Paradis G, O'Loughlin J (2022). A longitudinal study of change in substance use from before to during the COVID-19 pandemic in young adults. Lancet Reg Health Am.

[33] Faric N, Yorke E, Varnes L, Newby K, Potts HW, Smith L, Hon A, Steptoe A, Fisher A (2019). Title correction: Younger adolescents' perceptions of physical activity, exergaming, and virtual reality: Qualitative intervention development study. JMIR Serious Games.

[34] Shaw LA, Wünsche BC, Lutteroth C, Marks S, Callies R (2015). Challenges in virtual reality exergame design. User Interfaces 2015: Proceedings of the 16th Australasian User Interface Conference (AUIC 2015).

[35] O'Loughlin EK, Barnett TA, McGrath JJ, Consalvo M, Kakinami L (2019). Factors associated with sustained exergaming: Longitudinal investigation. JMIR Serious Games.

[36] Iqbal M Pokémon Go Revenue and Usage Statistics (2022). Business of Apps.

[37] Farič N, Potts HWW, Rowe S, Beaty T, Hon A, Fisher A (2021). Running app "Zombies, Run!" users' engagement with physical activity: A qualitative study. Games Health J.

[38] Ellis LA, Lee MD, Ijaz K, Smith J, Braithwaite J, Yin K (2020). COVID-19 as 'game changer' for the physical activity and mental well-being of augmented reality game players during the pandemic: Mixed methods survey study. J Med Internet Res.

[39] Li J, Theng Y, Foo S (2016). Effect of exergames on depression: A systematic review and meta-analysis. Cyberpsychol Behav Soc Netw.

[40] Cornish K, Fox G, Fyfe T, Koopmans E, Pousette A, Pelletier CA (2020). Understanding physical literacy in the context of health: a rapid scoping review. BMC Public Health.

